# Diagnosis of Fibrinous Concretions in the Heart

**Published:** 1855-09

**Authors:** B. W. Richardson


Diagnosis of Fibrinous Concretions in the Heart.—By Dr. B. W. Richardson.—In former papers, Dr. Richardson has endeavored to bring prominently forward the observations of some of the pathologists of the seventeenth and eighteenth centuries, who have regarded the formation of fibrinous concretions in the heart as a cause of death; and to combine with these observations such new information as modern science affords. It is now his object to speak of the diagnosis of fibrinous concretions in the heart. The formation of these concretions depends either on superfibrination of the blood, or on languidity of the circulation; and thus they are met with both in sthenic and asthenic conditions of the system. The general results are nearly the same in all cases; but the symptoms are modified in detail by the locality and size and mode of formation of the concretion. Where concretions are formed in the right side of the heart—their most usual situation—the general symptoms are those of arrest of the nutrition and general life of the body. The left side of the heart being imperfectly supplied, the arterial circulation is weakened; the pulse becomes small and intermittent; the surface, especially at the extremities, is cold; the veins are engorged; there is a great anxiety; the muscles become restless and powerless; the brain refuses to act; the mind wanders; the pupil dilates; and the acts of excretion are often performed unconsciously. A distressing and peculiar form of dyspnœa also presents itself; it occurs not because the respiration is checked, for the respiratory murmur is audible enough, but because no blood is passing into the lungs to be oxidized. Towards the close of this condition, emphysema of the lungs is very frequently present, and the physical signs of this lesion are a valuable corroboration of the presence of fibrinous concretions. The laborious character of the respiration depends on the deficiency in the supply of blood to the respiratory muscles and nervous centers. This description is applicable to cases in which death occurs in the course of forty-eight or seventy-two hours; but the symptoms are much varied by many causes. In some instances, death takes place almost instantaneously, in consequence of the sudden dislodgement of a previously formed concretion. In other instances, the concretion is not sufficient to entirely impede the circulation; here the symptoms are extended over several days, and exhaustion, and perhaps anasarca, present themselves. In a fourth class, the symptoms may extend over a long series of years, and at times may be in a great measure absent; death occurring at some time from their sudden re-appearance.

Fibrinous concretions on the left side of the heart are generally found in the ventricle, and at the root and arch of the aorta. When gradually laid down, they are denoted by certain sufficiently obvious symptoms:

1. There is an unusual tumultuous action of the heart; which Dr. Richardson believes to be a decided indication, in cases of suspected fibrinous concretion, that the deposit is taking place on the *left* side.
2. The lungs present, probably invariably, signs of congestion.
3. The dyspnœa is less distressing than in the cases formerly described.
4. The surface of the body has a dark congested aspect.
5. The body is cold, and not only restless, but convulsed.
6. There is a tendency to coma.
7. Cough is almost always present, accompanied with copious secretion, often tinged with blood, in the bronchial tubes.

When a favorable *point d'appui* is present, as in valvular disease, the concretion may seize on this, and suddenly arrest the circulation. In other cases, the concretion may undergo a gradual series of organic changes, and give rise to symptoms much resembling those of valvular obstruction—pulmonary lesions, dropsy, and in many cases hypertrophy. The symptoms of a concretion, whether in the right or in the left side, will be modified by the pre-existence of any organic lesion in the heart or vessels; as by feebleness at any one point of the walls of the heart; by valvular disease; and by dilatation of the heart or of the aorta.

Dr. Richardson has never been able to ascertain the presence of any physical sign absolutely diagnostic of fibrinous concretions. The wild tumultuous action of the heart, present when a concretion is formed in the left cavities, may be met with when there is no concretion. Very often, when the concretion is large, the only physical sign observed is, that the beat of the heart is weak and irregular, and its sound somewhat muffled. In other cases, where the concretion is small and hard, and firmly attached, a sound may be heard which cannot be, *per se*, distinguished from a valvular bruit. In forming the diagnosis of fibrinous concretion, the author trusts to the general outline and history of the symptoms; and with this view, in cases where concretion might be suspected, he inquires especially into the presence of hyperinosis of the blood, of any local inflammation, or of the absorption of a morbid poison; he also examines the general condition of the patient, and the state of the systemic circulation, and of the heart and lungs; and if in these he detects the special signs which he had already described, he regards concretions as being present. The author has derived most instruction from the diagnosis of fibrinous concretions in the estimation of prognosis, and in withholding useless or injurious plans of treatment. In croup especially, the operation of tracheotomy should depend on the determination of the question whether there was a concretion in the heart or not.—*Half Yearly Ab. Med. Sciences.*

